# Appraising the Quality of Patient Decision Aids for Brain and Heart Health: An Environmental Scan with a Health Equity Lens

**DOI:** 10.1016/j.cjco.2026.03.012

**Published:** 2026-03-27

**Authors:** Krystina B. Lewis, Sandhya Goge, Alda Kiss, Meg Carley, Ann Marie Julien, Gloria Higdon, Jess G. Fiedorowicz, Ian D. Graham, Amélie M. Achim, Rob Beanlands, Semhal Gessese, Sharon Straus, Jodi Edwards, Mathieu Bujold, Sheldon Tobe, Dawn Stacey

**Affiliations:** a School of Nursing, Faculty of Health Sciences, University of Ottawa, Ottawa, Ontario, Canada; b University of Ottawa Heart Institute, Ottawa, Ontario, Canada; c Foustanellas Endocrine and Diabetes Centre, The Ottawa Hospital, Ottawa, Ontario, Canada; d Ottawa Hospital Research Institute, Ottawa, Ontario, Canada; e Patient / Caregiver Partner, Ottawa, Ontario, Canada; f Faculty of Medicine, University of Ottawa, Ottawa, Ontario, Canada; g School of Epidemiology and Public Health, Faculty of Medicine, University of Ottawa, Ottawa, Ontario, Canada; h Department of Psychiatry and Neuroscience, Université Laval, Quebec City, Quebec, Canada; i VITAM Sustainable Health Research Centre, Quebec City, Quebec, Canada; j Unity Health, Toronto, Ontario, Canada; k Institute of Health Policy, Management, and Evaluation and Department of Medicine, University of Toronto, Toronto, Ontario, Canada; l School of Public Health, Université de Montréal, Montreal, Quebec, Canada; m Sunnybrook Health Sciences Centre, Toronto, Ontario, Canada; n Department of Medicine, Temerty Faculty of Medicine, University of Toronto, Toronto, Ontario, Canada

**Keywords:** patient decision aids, brain–heart conditions, health equity, shared decision-making, environmental scan, quality appraisal

## Abstract

**Background:**

New brain–heart clinical practice guidelines recommend provision of patient decision aids (PtDAs) to support people with or at risk of brain and heart conditions. We aimed to identify and appraise the quality of PtDAs addressing brain–heart conditions and their consideration of health equity.

**Methods:**

We conducted an online environmental scan to identify publicly available PtDAs. Two reviewers independently searched, extracted data, and appraised their quality using International Patient Decision Aid Standards (IPDAS) criteria, the Patient Education Materials Assessment Tool (PEMAT), and the PROGRESS-Plus framework for health equity.

**Results:**

Of 2549 resources identified, 51 PtDAs were eligible. Nine addressed a combined brain–heart decision, 36 addressed primarily cardiovascular conditions with brain implications, and 6 addressed primarily brain conditions with heart implications. Few focused on brain–heart conditions concomitantly. None addressed dementia, and only one addressed depression as a primary condition with cardiovascular risk addressed as a secondary consideration. The mean IPDAS essential criteria score was 5.8 of 7 (SD 1.5, range 1-7). For PEMAT-P (for printable materials), 91% PtDAs achieved an adequate understandability rating (≥ 70%), and 56% achieved an adequate actionability rating (≥ 70%). The most frequently reported PROGRESS-Plus healthy equity items were age (80%), gender/sex (45%), and socioeconomic status (39%).

**Conclusions:**

Brain–heart guidelines recommend PtDA use, but our environmental scan highlighted the need for more of these important interventions. Few PtDAs focused on brain and heart conditions concomitantly, none addressed dementia, and only one addressed depression, which frequently coexist with cardiac conditions. PtDAs also lacked actionable guidance within an equity-informed framework to support quality decisions and decision-making processes for all.

## Résumé

**Contexte:**

Les nouvelles recommandations cliniques relatives aux troubles cérébraux et cardiaques préconisent l'utilisation d'outils d'aide à la décision des patients (OAD) pour accompagner les personnes atteintes ou exposées à des risques de troubles cérébraux et cardiaques. Notre objectif était d'identifier et d'évaluer la qualité de ces OAD traitant des troubles sur l'axe cœur-cerveau, ainsi que la manière dont ils prennent en compte l'équité en matière de santé.

**Méthodologie:**

Nous avons mené une analyse contextuelle en ligne afin d’identifier les OAD accessibles au public. Deux évaluateurs ont, de manière indépendante, effectué des recherches, extrait des données et évalué leur qualité à l’aide des critères des normes internationales des aides à la décision destinées aux patients (IPDAS), de l’outil d’évaluation des supports d’information destinés aux patients (PEMAT) et du cadre PROGRESS-Plus pour l’équité en matière de santé.

**Résultats:**

Sur les 2 549 ressources identifiées, 51 OAD étaient éligibles. Neuf traitaient d’une décision combinée sur l'axe cœur-cerveau, 36 traitaient principalement de troubles cardiovasculaires ayant des implications cérébrales, et 6 traitaient principalement de troubles cérébraux ayant des implications cardiaques. Peu d'entre elles se concentraient simultanément sur les affections cœur-cerveau. Aucune ne traitait de la démence, et une seule traitait de la dépression en tant que trouble principal, le risque cardiovasculaire étant abordé comme une considération secondaire. Le score moyen des critères essentiels de l'IPDAS était de 5,8/7 (écart-type 1,5, intervalle 1-7). Pour le PEMAT-P (pour des documents imprimables), 91 % des OAD ont obtenu une note de compréhensibilité adéquate (≥ 70 %) et 56 % une note d'applicabilité adéquate (≥ 70 %). Les éléments de l'échelle PROGRESS-Plus relatifs à l'équité en matière de santé les plus fréquemment rapportés étaient l'âge (80 %), le genre/sexe (45 %) et le statut socio-économique (39 %).

**Conclusion:**

Les lignes directrices relatives à l’axe cœur-cerveau recommandent l'utilisation des OAD, mais notre analyse contextuelle a mis en évidence la nécessité de multiplier ces interventions importantes. Peu d'OAD se concentraient simultanément sur les troubles cérébraux et cardiaques, aucun ne traitait de la démence et un seul abordait la dépression, qui coexistent fréquemment avec les troubles cardiaques. Les OAD manquaient également de conseils concrets s'inscrivant dans un cadre tenant compte de l'équité pour soutenir des décisions et une prise de décision de qualité.

Over 6 million Canadians live with brain and heart conditions, and 90% have at least one risk factor for heart disease, stroke, or vascular cognitive impairment.^1^ Research shows the brain and heart are intrinsically interconnected, share the same risk factors, and often have cumulative effects.^1^ Brain–heart conditions refer to interconnected cardiovascular and neurologic and/or mental health conditions that share risk factors and influence one another’s outcomes, such as coronary artery disease and depression, atrial fibrillation and stroke, and heart failure and cognitive impairment. Equity-deserving groups—such as Indigenous peoples, racialized communities, women and gender-diverse individuals—are disproportionately affected due to barriers limiting access to resources and opportunities.^1^^,^^2^ Yet, these conditions are managed by different specialties, often resulting in fragmented healthcare experiences for people affected, and missed opportunities to consider them jointly in healthcare decisions.

In 2025, the first brain–heart clinical practice guidelines were developed, marking a critical step in addressing how coexisting brain–heart conditions are managed, communicated, and understood.^3^ They propose 9 evidence-based recommendations for the prevention and management of brain and heart conditions. One recommendation endorses patient decision aids (PtDAs), an evidence-based intervention, to support people with or at risk of brain–heart conditions in participating in shared decision-making. PtDAs may raise awareness of the brain–heart connection, communicate the potential impact of options on each body system, explicitly state that a decision needs to be made, provide balanced information on available options and their advantages and disadvantages, and support patients in clarifying their values. High-certainty evidence shows that when people use PtDAs, they are more knowledgeable about their options, have a clearer understanding of their values, and are more likely to participate in decision-making,^4^ with even greater benefits for equity-deserving groups.^5^^,^^6^

Brain–heart decision-making is particularly relevant for conditions such as dementia and depression, which are associated with cardiovascular disease and its treatments. Depression is both a risk factor for and a consequence of cardiovascular disease, and its management may involve pharmacologic therapies with important cardiovascular considerations. Dementia is similarly linked with vascular health and can complicate shared decision-making due to cognitive impairment, complex risk–benefit tradeoffs, and the need for caregiver involvement. Despite their clinical and decisional relevance, whether existing PtDAs adequately address dementia or depression within a brain–heart context remains unclear.

Although PtDAs exist for cardiac, neurologic, and mental health conditions, to what extent they consider the brain–heart connection is unclear. We sought to identify and appraise the quality of brain–heart focused PtDAs to determine which brain or heart connection they address, and report on their health equity considerations.

## Objectives

The objectives of this study were as follows: (i) to identify publicly available PtDAs addressing brain–heart conditions; (2) to appraise their quality using the International Patient Decision Aid Standards (IPDAS) essential criteria and the Patient Education Materials Assessment Tool (PEMAT), including understandability and actionability; and (iii) to assess the extent to which these PtDAs incorporate health equity considerations, using the PROGRESS-Plus framework. PROGRESS‑Plus is an equity framework encompassing place of residence, race/ethnicity/culture/language, occupation, gender/sex, religion, education, socioeconomic status, and Social capital, with additional “Plus” factors (eg, age, disability, or other characteristics associated with discrimination).

## Methods

### Study design

Our team of researchers, clinicians, and patient/caregiver partners co-conducted an environmental scan using online searches following the Preferred Reporting Items for Systematic Reviews and Meta-Analyses (PRISMA)^7^ and PRISMA-Equity 2012 Extension^8^ guidelines to identify and appraise brain–heart PtDAs.^9^^,^^10^

### Patient–caregiver partner involvement

To ensure relevance for PtDA users, 2 patient–caregiver partners (A.M.J., G.H.) contributed to the funding proposal, decisions about methods (eg, PtDA eligibility), executive meetings, and article preparation. Both patient–caregiver partners had lived experience with brain–heart conditions: A.M.J. is a patient with lived experience of a brain–heart condition, and G.H. is a caregiver for a person with a brain–heart condition. The patient and caregiver partners were purposively recruited through established collaborations with the first author (K.B.L.) and existing networks within the University of Ottawa Heart Institute. They helped focus the scan on issues meaningful to PtDA users, and provided insights into how findings would be understood and used, influencing recommendations. Their early, consistent, and meaningful engagement ensured that the study objectives were aligned with the real-world priorities, and enhanced their relevance. Ongoing and frequent communication fostered trust and a sense of shared ownership throughout this process.

### Eligibility criteria

We included PtDAs that (i) were freely available online; (ii) met IPDAS qualifying criteria; (iii) were published within 10 years to ensure current evidence; (iv) addressed brain/mental health and heart health decisions; and (v) were in any format (ie, written, audio, and/or visual) or language. Tools intended for clinicians only were excluded.

### Data sources and search strategy

We used the following data sources: PtDA repositories, Google searches, and a cardiovascular-focused PtDA systematic review^11^ (Table 1). Searches followed a standardized protocol using an advanced search function with various keyword combinations, limited to January 2015-April 2025. Non-English/French resources were translated using Google Translate (Google, Mountain View, CA).

**Table 1:** Sources of brain–heart patient decision aids (PtDAs)

Organization	Website
Ottawa Hospital Research Institute A-Z Inventory of Patient Decision Aids	https://decisionaid.ohri.ca/azinvent.php
AMC Decision Aids	https://deelkunde.nl/tools/decision-aids/ [in Dutch]
NHS England Decision support tools	https://www.england.nhs.uk/personalisedcare/shared-decision-making/decision-support-tools/
thuisarts Consultation Card Dutch DAs	https://www.thuisarts.nl/overzicht/keuzekaarten [in Dutch]
Sygehus Lillebælt Danish Alphabetical Library	https://sygehuslillebaelt.dk/afdelinger/stab-service-og-centre/center-for-faelles-beslutningstagning/beslutningsstottevaerktojer/alphabetical-library-of-developed-decision-helpers
Laval University Decision Box	https://www.boitedecision.ulaval.ca/en/
thuisarts Home Doctor Dutch DAs	https://www.thuisarts.nl/overzicht/onderwerpen [in Dutch]
Mayo Clinic DAs	https://carethatfits.org/tools/
MED-DECS	https://www.med-decs.org/en/
National Institute for Health and Care Excellence (NICE) DAs	https://www.nice.org.uk/about/nice-communities/nice-and-the-public/making-decisions-about-your-care/patient-decision-aids
Norwegian Inventory of PtDAs	https://www.helsenorge.no/samvalg [in Norwegian]
Patient+ DAs	https://www.keuzehulp.info/ [in Dutch]
University of Sydney DAs	https://www.sydney.edu.au/medicine-health/our-research/sydney-school-of-public-health.html
**Publications**	**Citation**
Cochrane Library Review of PtDAs 2024	https://www.cochranelibrary.com/cdsr/doi/10.1002/14651858.CD001431.pub6/full
European Journal of Cardiovascular Nursing Systematic Review of cardiovascular-focused PtDAs	https://doi.org/10.1093/eurjcn/zvaf103
**Search engines**	**Search terms**
Google and Google Scholar	Search 1: (HEART) "decision aid" AND cardiac not cancer; Search 2: (BRAIN) ("mental health" OR "stroke" OR "dementia" OR "cognitive impairment") AND "decision aid" not cancer.

AMC, Academic Medical Center; DA, decision aids; NHS, National Health Service;.

Google and Google Scholar (Google, Mountain View, CA).

Two reviewers (A.K., M.C.) independently searched Google and Google Scholar, using key terms (Table 1). English-only searches were conducted in private Chrome 2025 windows to prevent previous searches’ catching results.^12^ Results were reviewed until all were examined or 50 consecutive irrelevant results were identified.^13^ Browsers were cleared and cookies disabled before each search to avoid introducing bias; Web site addresses were recorded in Excel (Microsoft, Redmond, WA). Two reviewers (A.K., M.C.) independently screened resources for eligibility and removed duplicates. Disagreements were resolved with a third reviewer (K.B.L.).

### Data extraction

Two reviewers (A.K., M.C., or S.G.) independently extracted data from eligible PtDAs using a standardized spreadsheet including the following: (i) source (eg, inventory/search source, URL, date accessed); (ii) resource characteristics (eg, title, format, country, primary decision (ie, heart, brain, combined) and degree of integration of other organ per 7 IPDAS qualifying criteria, PtDA title, and embedded knowledge test, if present; (iii) the PEMAT for understandability/actionability; and (iv) PROGRESS-Plus elements known to impact health equity. Reviewers (A.K., K.B.L., M.C., S.G.) piloted the extraction sheet on 12 PtDAs, reconciled differences, made minor changes (eg, the extent to which the other organ/condition is mentioned), and retained pilot data.

### Appraisal frameworks and instruments

The IPDAS are internationally developed, consensus-based criteria designed to improve the quality, transparency, and trustworthiness of PtDAs. The IPDAS qualifying criteria define the minimum standards required to be considered a PtDA.^14^ These criteria include clear description of the health condition, explicit mention of the decision and the options, target audience, balanced presentation of the positive and negative features of the options and outcomes, and values clarification. These criteria have been applied widely and operationalized across diverse clinical conditions, decision contexts, and health systems. If the IPDAS qualifying criteria were met, we then appraised the PtDAs against the IPDAS essential criteria (7 core criteria with 3 additional criteria for screening decisions) for minimizing risk of making a biased decision, with no minimal score for adequacy. Specifically, these IPDAS essential criteria aim to reduce the risk of harmful bias by assessing whether a PtDA is based on the best available evidence, reports user involvement in development, presents negative/positive features of options in a balanced manner, and includes key elements such as funding sources, evidence citations, publication date, and update policy.

The PEMAT is a reliable and validated instrument used to assess resources for 2 distinct domains—understandability and actionability. Understandability refers to whether people of diverse backgrounds and levels of health literacy can process and explain the key messages (eg, clarity of language, organization, use of visuals). Actionability refers to whether the material clearly identifies actions users can take and provides sufficient guidance to support those actions (eg, specific steps, prompts, or tools).^15^ PEMAT-P applies to printable materials (eg., brochures, PDFs); and PEMAT-A/V applies to audiovisual materials (eg, videos, multimedia).

PROGRESS-Plus is a framework for identifying factors contributing to health inequities. PROGRESS stands for: place of residence; race/ethnicity/culture/language; occupation; gender/sex; religion; education; socioeconomic status; and social capital. “Plus” adds 3 context-specific factors that may further contribute to health inequalities: (i) personal characteristics associated with discrimination; (ii) features of relationships; and (iii) time-dependent relationships.^16^

### Quality appraisal

Two reviewers (A.K., S.G., or M.C.) independently appraised resources in 3 stages; a third (K.B.L. or M.C.) resolved discrepancies. First, we appraised resources using the IPDAS essential criteria, scored as “present,” “absent,” or “unclear.” An “unclear” rating was used when criteria were not explicitly reported or could not be ascertained from the PtDA.^17^ For analytic purposes, criteria rated as unclear were treated as not meeting the criterion and were not counted toward the total IPDAS score. Second, we scored understandability (PEMAT-P, 17 items; PEMAT-AV, 4 items) and actionability (PEMAT-P, 7 items; PEMAT-AV, 4 items).^15^ Ratings given were 0 (disagree), 1 (agree), or Not Applicable;^15^^,^^18^ discrepancies were resolved by a third reviewer (K.B.L. or M.C.). Third, we rated PROGRESS-Plus items as either present or absent; discrepancies were resolved by a third reviewer (K.B.L. or M.C.).

### Data analysis

For analytic purposes, PtDAs were categorized according to how brain and heart considerations were framed within the decision. PtDAs that explicitly addressed interrelated cardiovascular, brain and/or mental health considerations were classified as combined brain–heart conditions (eg, atrial fibrillation and stroke, heart failure and cognition). Cardiovascular- focused conditions included those in which heart disease was the primary focus but that incorporated brain or mental health considerations as risks, outcomes, or contextual factors (eg, coronary revascularization with periprocedural stroke risk or depression following cardiac events). Brain or mental health–focused conditions included those in which brain or mental health conditions were primary, with cardiovascular considerations incorporated as comorbidities, contraindications, or treatment risks (eg, psychosis or depression treatments with cardiac side effects).

We used descriptive statistics to report and synthesize data of eligible PtDAs, their brain–heart connection, IPDAS criteria, and PROGRESS-Plus determinants. PEMAT scores were calculated using the proportion of applicable items met (excluding Not Applicable), as not all items were relevant for each PtDA. PEMAT scores ≥ 70% indicate adequate understandability and actionability of a PtDA.^15^

## Results

**Figure 1:**
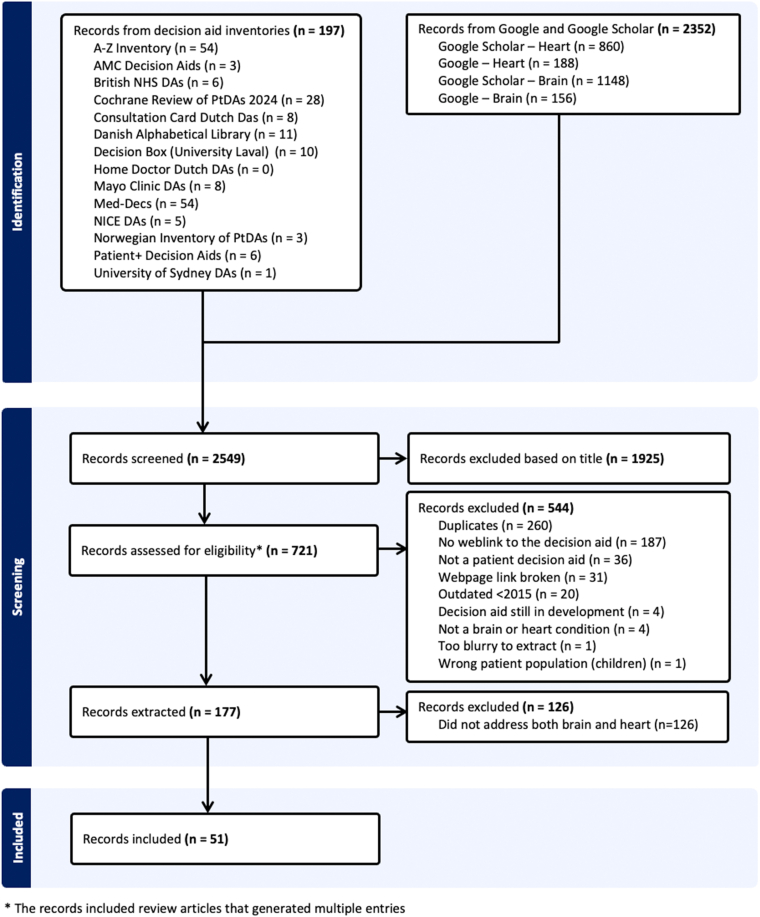
Preferred reporting items for systematic reviews and meta-analyses (PRISMA) diagram. AMC, Academic Medical Center; DAs, decision aids; NHS, National Health Service; NICE, National Institute for Health and Care Excellence; PtDAs, patient DAs. Google, Google Scholar (Google, Mountain View, CA)

Searches identified 2549 records—197 through PtDA inventories, and 2352 through Google/Google Scholar searches (Fig. 1). After screening and de-duplication, 51 PtDAs were included.

PtDAs spanned 8 countries: the US (61%); the United Kingdom (10%); Canada (8%); the Netherlands (8%); Japan (6%); Germany (4%); Taiwan (2%); and Australia (2%; Table 2). Most PtDAs were available in English only (88%). Eleven (22%) were available in multiple languages, most commonly Spanish, with a small number available in French, Dutch, Japanese, or German. PtDAs available in multiple languages were primarily developed in the US, Canada, and the Netherlands (Table 2). PtDAs were available in various formats—static online (47%), interactive online plus print (25%), interactive online (16%), static online and video (8%), and interactive online with print and video options (4%). PtDAs were designed for use before consultation (92%), during consultation (6%), or unclear timing (2%). Most (61%) were updated within the past 5 years, and 27% between 2015-2019; 12% had no update date. Nonprofit medical organizations were most often the developer (55%).

**Table 2:** Summary of included patient decision aid (PtDA) characteristics (n = 51)

Characteristics of included PtDAs	Value
Topic	
Combined (cardiac and brain) as primary condition	
Stroke and cardiovascular disease prevention – medication options	8 (16)
Patent foramen ovale and stroke	1 (2)
Cardiovascular as primary condition	
Atrial fibrillation and risk for stroke – medication options	10 (20)
Procedures for coronary artery disease (revascularization) and risk of stroke	7 (14)
Aortic valve replacement and risk for stroke	6 (12)
Atrial fibrillation and risk for stroke – medication and procedural options	5 (10)
Left ventricular assist device and risk for stroke	4 (8)
Peripheral arterial disease and risk of stroke	2 (4)
Heart failure and noninvasive vs invasive treatments and risk of stroke	1 (2)
Carotid artery stenosis and risk of stroke	1 (2)
Brain/mental health as primary condition	
Mental health treatment and risk of heart complications (eg, arrhythmias, high blood pressure)	5 (10)
Depression and cardiovascular disease	1 (2)
Year of last update	
2015–2019	14 (28)
2020–2025	31 (61)
Not reported	6 (12)
Country	
US	31 (61)
United Kingdom	5 (10)
Canada	4 (8)
Netherlands	4 (8)
Japan	3 (6)
Germany	2 (4)
Taiwan	1 (2)
Australia	1 (2)
Languages	
English	45 (88)
Spanish	9 (18)
Dutch	3 (6)
Japanese	3 (6)
French	2 (4)
German	1 (2)
Developer type	
Medical organization—nonprofit	28 (55)
Independent research team	11 (22)
National health organization	7 (14)
Medical school	3 (6)
Medical organization—for profit	1 (2)
Provincial health organization	1 (2)
Format	
Static online (ie, PDF to print)	24 (47)
Interactive online with option to print	13 (26)
Interactive online only	8 (16)
Static online and video	4 (8)
Interactive online with option to print, and video	2 (4)
Timing of use	
Before consult	47 (92)
During consult	3 (6)
Unclear	1 (2)
Estimated length	
Static online (pages; n = 43)	13.3 (14.5), 9, [1-69]
Interactive online (pages; n = 23)	10.0 (7.9), 6, [4-33]
Video (min; n = 6)	13.2 (9.6), 12.8, [1.7-26.3]

Values are n (%), or mean (standard deviation), median [range].

### Brain–heart connection of included PtDAs

**Figure 2:**
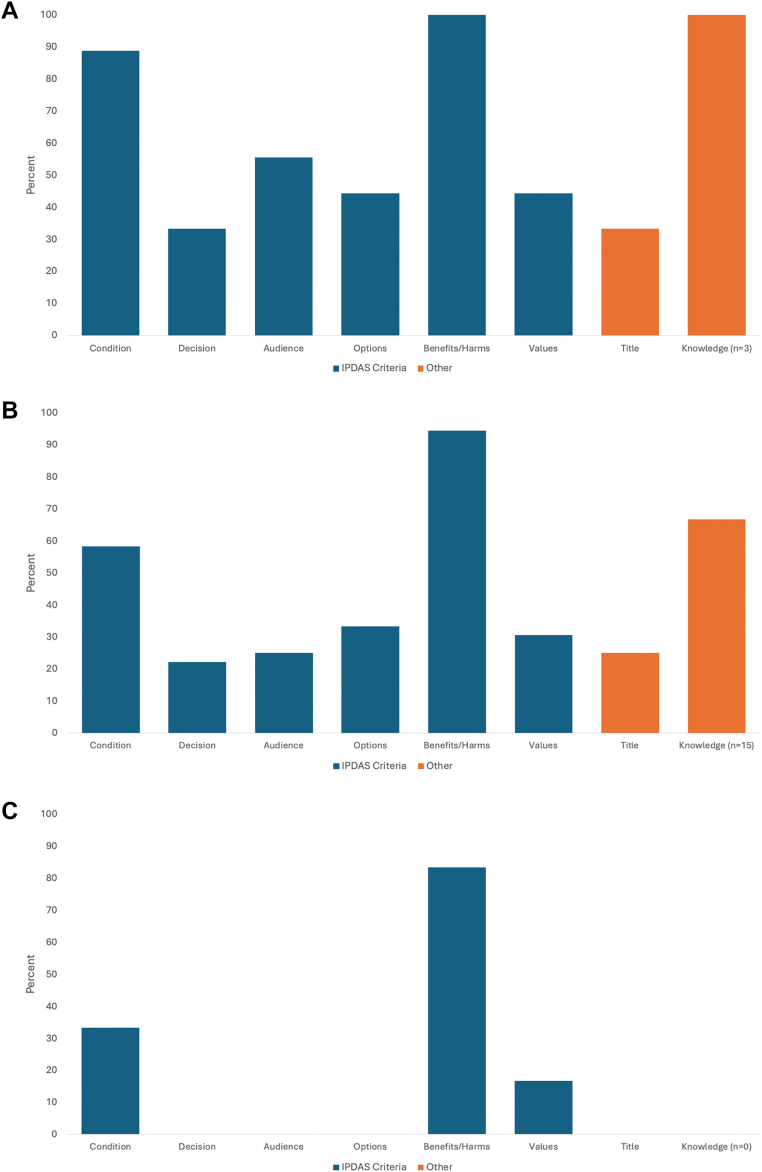
Location of brain–heart considerations within international patient decision aid standards (IPDAS) qualifying criteria, the patient decision aid (PtDA) title, and knowledge test (if applicable). The proportion of brain–heart considerations that were explicitly incorporated within each IPDAS criterion for PtDAs focused on (**A**) combined cardiac and brain as primary condition (n = 9), (**B**) cardiovascular disease as a primary condition(n = 36), or (**C**) brain/mental health as a primary condition (n = 6). All PtDAs met all IPDAS qualifying criteria. The figure does not represent whether IPDAS qualifying criteria were met, but where brain–heart connections were explicitly mentioned within each criterion. The 7 IPDAS qualifying criteria are as follows: (1) describes the health condition; (2) explicitly states the decision to be considered; (3) identifies the target audience; (4) lists options, including if relevant, “wait and see” (eg, making no change, doing nothing); (5) describes positive features of options (benefits); (6) describes negative features of options (harms); (7) asks patients to think about which positive and negative features of options matter most to them OR describes what it is like to experience the consequences of options (physical, psychological, social).

Of 51 PtDAs, 9 (18%) addressed decisions affecting both the brain and heart (Table 2; Supplemental Table S1); 8 PtDAs (16%) addressed stroke and cardiovascular disease prevention; and 1 (2%) addressed patent foramen ovale closure and stroke. Figure 2 illustrates the proportion of PtDAs in which brain–heart considerations were explicitly integrated within each of the IPDAS qualifying criteria. Brain–heart connections appeared most under benefits/harms (100%), condition (89%), target audience (56%), and all knowledge tests available (100%; Fig. 2A); they were least reported in options (44%), values clarification (44%), index decision (33%), and title (33%).

Thirty-six PtDAs (71%) addressed cardiovascular disease as the primary condition, with brain health (ie, risk of stroke) as secondary. Common cardiovascular conditions included atrial fibrillation (42%), coronary artery disease (19%), and aortic valve replacement (17%), with risk of stroke as the main secondary condition for all. Brain–heart connections appeared most under benefits/harms (94%), knowledge test (67%), and condition (58%; Fig. 2B), and least in target audience (25%), title (25%), and index decision (22%). Only one PtDA (2%) addressed screening for stable ischemic heart disease.

Six PtDAs (12%) focused on brain and/or mental health conditions, with heart mentioned as a secondary focus (Fig. 2C). Brain and/or mind conditions addressed were mental health treatment in general (83%) and depression (17%), with the risk of cardiovascular complications as a secondary focus. Brain–heart connections appeared most under benefits/harms (83%), condition (33%), and values (17%), and were not reported in the title, decision, knowledge test, target audience, or options categories.

### IPDAS quality appraisal

The mean IPDAS essential criteria score was 5.8/7 (standard deviation [SD] 1.5, range 1-7; Table 3; Supplemental Table S2). Twenty-four PtDAs (47%) met all 7 criteria. Most of the criteria met were negative and positive features in a balanced manner (96%), best available evidence (88%), publication date (86%), and how potential users were involved in development (84%). Notably, descriptions of involved patients were vague. Few reported whether patients had lived experience with the condition(s), and none specified involving equity-deserving groups. The least-met criteria were complete citations to evidence (80%), funding source (76%), and update policy (67%). Regardless of IPDAS scoring, PtDAs addressed the brain–heart connection most often in the benefits and harms criteria. Those with the highest IPDAS scoring were least likely to mention it in the title and decision. Those with lowest IPDAS scoring were least likely to mention it in the decision, options, and values areas.

**Table 3:** Iinternational patient decision aid standards (IPDAS) and patient education materials assessment tool (PEMAT) scores

	IPDAS essential criteria, of 7 (n = 51)	PEMAT-P (n = 45)∗		PEMAT-AV (n = 6)	
Measure		Understandability (0–100)	Actionability (0–100)	Understandability (0–100)	Actionability (0–100)
**All PtDAs**					
Mean	5.8	86.2	73.3	91.9	95.8
SD	1.5	11.2	21.4	12.9	10.2
Median	6	87.5	83.3	96.2	100
Range	1–7	52.9–100	0–100	66.7–100	75.0–100
Proportion meeting all 7 criteria	24 (47.1%)	—	—	—	—
Proportion ≥ 70%	—	41 (91.1%)	25 (55.6%)	5 (83.3%)	6 (100%)
**Combined cardiac and brain as primary condition**					
Mean	5.2	82.9	59.3	—	—
SD	2.3	12.4	27.0	—	—
Median	6	84.6	66.7	—	—
Range	1–7	52.9–93.8	0–83.3	—	—
Proportion meeting all 7 criteria	4 (44.4%)	—	—	—	—
Proportion ≥ 70%	—	8 (88.9%)	3 (33.3%)	—	—
**Cardiovascular as primary condition**					
Mean	5.8	86.9	75.1	91.9	95.8
SD	1.4	11.3	18.4	12.9	10.2
Median	6	88.2	83.3	96.2	100
Range	2–7	57.1–100	33.3–100	66.7–100	75.0–100
Proportion meeting all 7 criteria	17 (47.2%)	—	—	—	—
Proportion ≥ 70%	- - -	29 (90.6%)	19 (59.4%)	5 (83.3%)	6 (100%)
**Brain / mental health as primary condition**					
Mean	6.5	88.2	90.1	—	—
SD	0.6	8.0	15.9	—	—
Median	6.5	85.2	96.9	—	—
Range	6-7	82.4-100	66.7-100	—	—
Proportion meeting all 7 criteria	3 (50.0)	—	—	—	—
Proportion ≥ 70%	—	4 (100%)	3 (75.0%)	—	—

PEMAT-AV, PEMAT—audio visual materials; PEMAT-P, PEMAT—printable materials; SD, standard deviation.

∗ PEMAT not conducted on non-English French PtDAs.

PEMAT quality appraisal

Of 51 PtDAs, 45 (88%) were appraised with PEMAT-P, and 6 (12%) with PEMAT-AV (Table 3; Supplemental Table S2). We did not appraise 6 non-English PtDAs (12%).

**Figure 3:**
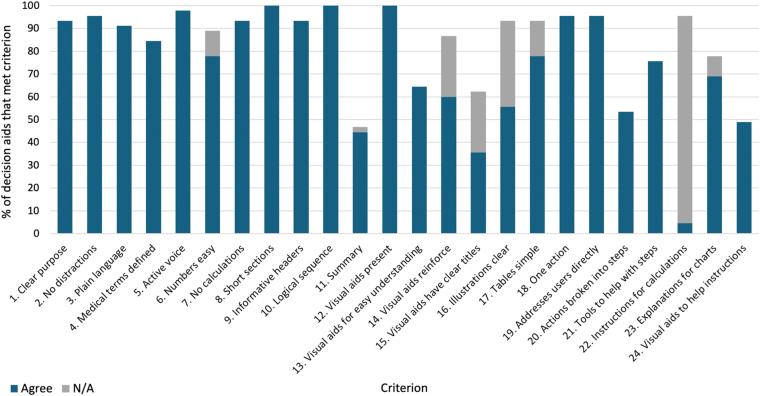
Patient education materials assessment tool—printable materials (PEMAT-P) item-level agreement across printable patient decision aids (PtDAs; n = 45). **Bars** represent the proportion of PtDAs rated as “Agree” for each PEMAT-P item, with values calculated after excluding items marked not applicable (N/A), in accordance with PEMAT scoring guidance. Understandability items (items 1–17) assess clarity, organization, and presentation of information. Actionability items (items 18–24) assess whether the material clearly identifies actions users can take and provides sufficient guidance to support those actions. Higher percentages indicate that a greater proportion of PtDAs met the specified criterion. **Blue bars** represent the percentage of PtDAs that met the PEMAT criterion, defined as items rated “Agree” by reviewers. **Grey bars** represent the percentage of PtDAs for which the PEMAT item was not applicable (N/A) and was therefore excluded from the scoring.

For PEMAT-P (n = 45), the mean understandability score was 86.2 of 100 (SD 11.2, range 52.9-100), and 41 PtDAs (91%) achieved an adequate understandability rating of ≥ 70% (Table 3). Most categories met the understandability criteria: short sections (100%), logical sequence (100%), visual aids present (100%), and active voice (98%). The least-met understandability criteria were: provides a summary (44%) and visual aids have clear titles/captions (48%; Fig. 3). The mean actionability rating was 73.3 of 100 (SD 21.4, range 0-100); only 25 PtDAs (56%) achieved an adequate actionability score of ≥ 70% (Table 3). Most met actionability criteria: identified at least one action a user can take (96%), addressed users directly (96%), and provided tangible tools to help users take action (76%). Least-met actionability criteria were as follows: explained how to use charts/graphs/tables/diagrams to take an action (76%); provided instructions for calculations (50%); and used visual aids to make it easier to act on instructions (49%). PtDAs that focused primarily on brain conditions scored the highest for understandability (100%) and actionability (75%).

Six PtDAs were video-based, with a mean PEMAT-AV understandability score of 91.9 of 100 (SD 12.9, range 66.7-100). Five PtDAs (83%) achieved an adequate understandability rating of ≥ 70%. The mean actionability score was 95.8 of 100 (SD 10.2, range 75-100); all PtDAs with videos achieved an adequate actionability rating of ≥ 70% (Table 3).

### PROGRESS-Plus for health equity

The most frequently reported PROGRESS-Plus characteristics were gender/sex (45%), socioeconomic status (39%), and social capital (27%). The least-addressed characteristics were race (including one Indigenous; 12%), education (10%), place of residence (6%), and religion (6%; Supplemental Table S3). For Plus (1), personal characteristics associated with discrimination were the most frequently addressed (80%), with age mentioned most.

## Discussion

We identified 51 PtDAs addressing a range of brain–heart conditions. Although some PtDAs directly addressed interlinked brain–heart conditions, the majority focused on the relationship between cardiovascular conditions and stroke. Most PtDAs met IPDAS essential and PEMAT understandability thresholds, whereas only half met PEMAT actionability criteria. Few PtDAs incorporated characteristics related to health equity, beyond age, and sex and/or gender. Together, these findings suggest that clear opportunities are available to (i) be more inclusive of other brain conditions that concomitantly exist with cardiovascular conditions, and vice-versa; (ii) improve how decisions and the options are framed to consider the complexities between these 2 organ systems; (iii) be more actionable; and (iv) integrate more equity-focused considerations known to influence decision-making and outcomes. Our findings lead us to the following points of discussion.

The presence of multiple PtDAs for the same brain–heart conditions reflects both strong interest and sustained effort in developing interventions to support patient involvement in health decisions that fit the local context in which they were developed and where they are intended to be used. However, some conditions were not represented. We did not identify PtDAs that addressed cognitive impairment (or a variant) either as a primary condition or in relation to a cardiac condition. This gap is significant, as cognitive impairment commonly co-occurs with heart conditions in aging, due to shared risk factors.^19^ Modifying cardiovascular risk factors may influence the onset or progression of some types of dementia and other forms of vascular cognitive impairment. The new brain–heart clinical practice guidelines recommend dementia screening for early detection and prevention in people with atrial fibrillation, further underscoring the need for PtDAs, because screening for dementia is complex, with pros and cons valued differently based on individual experience and context.^20^^,^^21^ Some may value early identification of cognitive changes to enable timely implementation of evidence-based strategies (eg, healthy lifestyle behaviours, risk-factor modification, medication) that may slow progression. Others may worry about potential consequences of early detection, including loss of independence (eg, losing their drivers’ license) and social stigma, which could affect their quality of life.^22^, ^23^, ^24^ A person’s health status and perceived illness risk can affect their acceptance of early dementia screening.^20^ This finding is particularly relevant in this context when dementia coexists with a cardiac condition. Hence, involving people and their families in these decisions is important, and PtDAs can support this effort.

The new brain–heart clinical practice guidelines also recommend screening for depression in people with heart failure.^3^ Few PtDAs focusing on major depression are available, representing another gap. Major depression is a known contributor to poor outcomes in people with cardiac conditions,^25^ and its treatments may influence cardiometabolic risk.^26^ This issue is also preference-sensitive, as some patients may value improvements in mental health and quality of life, whereas others may have concerns about associated stigma. Specific conditions aside, few PtDAs integrated brain–heart considerations across IPDAS qualifying criteria, such as naming the decision as one that impacts both the brain and heart, listing the options, and listing how a user might think of both the brain and heart when clarifying their values. Future PtDA development should ensure that brain–heart considerations are addressed and incorporated with options and outcomes, and that values clarification explicitly prompts users to consider tradeoffs relevant to both brain and heart health. As noted by IPDAS, no single threshold defines a “good-quality” PtDA. Rather, the essential criteria are intended to identify strengths and gaps that may affect a biased influence on decisions. Hence, rather than recommending exclusive use of highest-scoring PtDAs, we suggest a democratic selection process, in which end-users, clinicians, organizations, and developers jointly select, use, and maintain tools that meet core quality standards while addressing gaps identified in this appraisal.

To maximize the impact PtDAs can have on brain–heart decisions, they must be more action-oriented. In our study, half of printed PtDAs met actionability ratings of ≥ 70%; whereas all videos did. Interestingly, PtDAs that focused primarily on brain conditions scored the highest on actionability (3 of 4; 75%). Developers of PtDAs for complex, interconnected brain–heart conditions must consider their format and structure to include concrete steps, guidance, and graphics that empower patients to act on the information presented, and prioritize competing decisional needs, particularly in a context in which potential users may experience cognitive difficulties.^27^^,^^28^ Doing so is challenging in healthcare systems that are not designed for integrated care of coexisting conditions.^1^^,^^29^

Assessment using the PROGRESS-Plus framework revealed that few PtDAs addressed elements of health equity known to influence health outcomes, with race, education, and place of residence mentioned least often. Although sex and gender considerations were most frequently incorporated, the absence of sex- or gender-specific PtDAs indicates an opportunity to develop more tailored tools that reflect sex-based differences in brain–heart risk and disease, as well as gendered disparities in care, health behaviours, and lived experiences.^11^ The same need applies for culturally adapted PtDAs.^30^ Researchers’ awareness and sensitivity to the particular needs of equity-deserving groups and their meaningful engagement in PtDA development and evaluation may promote inclusion of broader factors that influence health, preferences, and decision-making, a practice recommended by IPDAS.^14^ PtDAs promote health equity, as they are designed to provide equitable access to information, build trust, minimize implicit bias, and support conversations that incorporate people’s values, concerns, and life circumstances. To promote equity-informed decision-making, future PtDAs should more systematically acknowledge social and contextual factors known to influence preferences, access to health services, and decision-making outcomes. Meaningful engagement of equity-deserving groups during PtDA development may support more inclusive content and enhance relevance across diverse populations. Developers grounded in health equity may also foster more inclusive processes and tools.

Potential downsides to explicitly addressing all PROGRESS-Plus characteristics should be considered. Factors associated with discrimination may be difficult for individuals to disclose, particularly in healthcare environments in which fears of stigma, judgment, or unintended consequences persist.^31^^,^^32^ Equity-responsive PtDA design therefore requires a careful balance between inclusivity and respect for patient autonomy. Approaches such as using inclusive language, normalizing diverse life circumstances, and offering optional or reflective prompts rather than direct requests for disclosure of information may support equity-informed decision-making while preserving trust and safety.

### Strengths and limitations

The search strategy of using Web sites was comprehensive and allowed us to find publicly available sources that might not have been found through traditional database searches. Two screeners independently identified PtDAs using clean browsers and extracted data. However, due to changing Web site content, some previously published resources may be missing or relocated, impacting reproducibility. Given that the search was done with English terms only, non-English language PtDAs may have been missed.

## Conclusion

We identified 51 PtDAs for brain–heart interrelated conditions. Few embraced these co-occurring conditions concomitantly. Significant gaps were present for emerging brain–heart linked conditions (eg, dementia and heart disease, depression and heart disease). Publicly available PtDAs could be improved by embedding actionable guidance within equity-informed frameworks to support quality decisions and decision-making processes.

## Ethics Statement

This study was an environmental scan of publicly available patient decision aids. No human participants were recruited, and no identifiable personal data were collected. As such, institutional research ethics board approval was not required.

## Patient Consent

The authors confirm that patient consent is not applicable to this article. This was an environmental scan of publicly available patient decision aids and no participants were recruited; therefore, no institutional review board review was necessary.

## Funding Sources

This work is funded by Canada First Research Excellence Fund via the Brain-Heart Interconnectome (BHI 23 – 124). K.B.L. is supported by a Heart and Stroke New Investigator Award NI-24-0036356.

## Disclosures

J.E. and S.T. chaired the Brain–Heart Clinical Practice Guideline Group. K.B.L. and D.S. are coauthors of the Brain–Heart Clinical Practice Guidelines. The other authors have no conflicts of interest to disclose.

## Footnotes

To access the supplementary material accompanying this article, visit *CJC Open* at https://www.cjcopen.ca/ and at https://doi.org/10.1016/j.cjco.2026.03.012
