# Supplementary material for: Appraising the Quality of Patient Decision Aids for Brain and Heart Health: An Environmental Scan with a Health Equity Lens
Source: CJC Open. 2026 Mar 27;8(7):897–907. doi: 10.1016/j.cjco.2026.03.012 (PMC13386744; doi:10.1016/j.cjco.2026.03.012)
Supplement: Supplementary Table [file mmc1.docx]

Supplemental Table S1. Characteristics of Included PtDAs (n=51 PtDAs)

| **ID. Title of PtDA (Year of last update). Availability.** | **Options** | **Developer, Country** | **Language(s)** | **Format/ Length** | **Timing of use** |
| --- | --- | --- | --- | --- | --- |
| **Combined cardiac and brain as primary condition (n=9)** | | | | | |
| **Stroke and Cardiovascular Disease Prevention – Medication Options (n=8 PtDAs)** | | | | | |
| 1. Statins: Should I Take Them to Prevent a Heart Attack or Stroke? (2024). Available [here](https://decisionaid.ohri.ca/AZsumm.php?ID=1080). | Statins vs No statins | Healthwise, USA | English | Online, Print (6 pages) | Before consult |
| 2. Statins: Are these cholesterol-lowering drugs right for you? (2024). Available [here](https://www.mayoclinic.org/diseases-conditions/high-blood-cholesterol/in-depth/statins/art-20045772). | Statins vs No statins | Mayo Clinic, USA | English | Online (1 page) | Before consult |
| 3. Statin side effects: Weigh the benefits and risks (2025). Available [here](https://www.mayoclinic.org/diseases-conditions/high-blood-cholesterol/in-depth/statin-side-effects/art-20046013). | Statins vs No statins | Mayo Clinic, USA | English | Online (1 page) | Before consult |
| 4. Should I take a statin? (2023). Available [here](https://www.nice.org.uk/guidance/ng238/resources/patient-decision-aid-pdf-243780159). | Statins vs No statins | NICE, UK | English | Print (12 pages) | Before consult |
| 5. Aspirin: Should I Take Daily Aspirin to Prevent a Heart Attack or Stroke? (2024). Available [here](https://decisionaid.ohri.ca/AZsumm.php?ID=1319). | Aspirin vs No aspirin | Healthwise, USA | English | Online, Print (6 pages) | Before consult |
| 6. High Blood Pressure: Should I Take Medicine? (2024). Available [here](https://decisionaid.ohri.ca/AZsumm.php?ID=1012). | Medicine vs Lifestyle changes | Healthwise, USA | English | Online, Print (6 pages) | Before consult |
| 7. How do I control my blood pressure? Lifestyle options and choice of medicines (2019). Available [here](https://www.nice.org.uk/guidance/ng136/resources/patient-decision-aid-pdf-6899918221). | Do nothing vs Lifestyle changes vs Medicines | NICE, UK | English | Print (10 pages) | Before consult |
| 8. What can you do to reduce your risk of having a heart attack or stroke? (NR). Available [here](https://jmir.org/api/download?alt_name=cardio_v6i1e34142_app1.xlsx&filename=1f8dfda6a907e048b73ba3c89d919330.xlsx). | Lifestyle changes, Supplements, Blood pressure medication, Cholesterol medication, Aspirin | Carissa Bonner et al. University of Sydney, Australia | English | Print (1 page) | Before consult |
| **Patent foramen ovale and Stroke (n=1 PtDA)** | | | | | |
| 9. Patent foramen ovale closure, antiplatelet therapy or anticoagulation therapy alone for management of cryptogenic stroke? A clinical practice guideline (2018). Available [here](https://www.bmj.com/content/362/bmj.k2515). | Patent foramen ovale closure vs Anticoagulants vs Antiplatelets | Ton Kuijpers, Netherlands | English | Online, Print (11 pages) | During consult |
| **Cardiovascular as primary condition (n=36)** | | | | | |
| **Atrial Fibrillation and Risk for Stroke – Medication Options (n=10 PtDAs)** | | | | | |
| 10. Atrial Fibrillation: Should I Take an Anticoagulant to Prevent Stroke? (2024). Available [here](https://decisionaid.ohri.ca/AZsumm.php?ID=1043). | Anticoagulant vs No anticoagulant | Healthwise, USA | English | Online, Print (6 pages) | Before consult |
| 11. Anticoagulation Choice (NR). Available [here](https://carethatfits.org/anticoagulation-choice/). | Anticoagulant vs No anticoagulant; Warfarin vs DOACs | Mayo Clinic, USA | English | Online (6 pages) | During consult |
| 12. Standford guide to Afib Stroke Prevention (NR). Available [here](https://afibguide.com/). | Anticoagulant vs No anticoagulant | Standford University, USA | English, Spanish | Online (4 pages), Print (2-page worksheet), and Video (4 min) | Before consult |
| 13. Shall I Take Oral Anticoagulants to Prevent Stroke if I Have AF (NR). Available [here](https://ndownloader.figstatic.com/files/42109549). | Anticoagulant vs No anticoagulant | Taipei Veterans General Hospital, Taiwan | English | Print (9 pages) | Before consult |
| 14. Atrial fibrillation guideline support tool (NR). Available [here](https://pmc.ncbi.nlm.nih.gov/articles/instance/4708062/bin/NIHMS741668-supplement-Supplementary_Material.pdf). | No anticoagulant vs Aspirin vs Warfarin | University of Cincinnati, Mark Eckman, USA | English | Print (25 pages) | Before consult |
| 15. RAPID AFib Decision Aid (2023). Available [here](https://rapiddecisionaids.ca/). | No anticoagulant vs Warfarin vs DOACs | University of Calgary, Justin Baers, Canada | English | Online (33 pages) | Before consult |
| 16. AFib and Moderate Risk for Stroke (2017). Available [here](https://www.cardiosmart.org/assets/decision-aid/afib-and-moderate-risk-for-stroke). | Warfarin vs DOACs | American College of Cardiology, USA | English, Spanish | Print (5 pages), Video (12 min) | Before consult |
| 17. AFib and High Risk for Stroke (2017). Available [here](https://www.cardiosmart.org/assets/decision-aid/afib-and-high-risk-for-stroke). | Warfarin vs DOACs | American College of Cardiology, USA | English, Spanish | Print (5 pages), Video (13 min) | Before consult |
| 18. Atrial Fibrillation: Which Anticoagulant Should I Take to Prevent Stroke? (2022). Available [here](https://www.healthlinkbc.ca/healthwise/atrial-fibrillation-which-anticoagulant-should-i-take-prevent-stroke). | Warfarin vs DOACs | Healthwise, USA | English | Online, Print (6 pages) | Before consult |
| 19. Patient / Healthcare Professional Decision Aid: ATRIAL FIBRILLATION Medicines to help reduce your risk of a stroke – What are the options? (2016). Available [here](https://www.northyorkshireandyorkformulary.nhs.uk/docs/BNF/02/Atrial%20fibrillation%20patient%20decision%20aid.pdf). | Warfarin vs DOACs | Vale of York Clinical Commissioning Group & York Teaching Hospitals NHS Foundation Trust, UK | English | Print (7 pages) | Before consult |
| **Atrial Fibrillation and Risk for Stroke – Medication and Procedural Options (n=5 PtDAs)** | | | | | |
| 20. A Decision Aid for AFib Stroke Prevention for Patients with Atrial Fibrillation (2017). Available [here](https://www.cardiosmart.org/topics/atrial-fibrillation/assets/decision-aid/afib-and-very-high-risk-for-stroke). | Treatment vs No treatment; Warfarin vs. DOAC; Blood thinner vs. Closure device | American College of Cardiology, USA | English, Spanish | Print (9 pages), Video (22 min) | Before consult |
| 21. Making a decision about: further treatment for atrial fibrillation (AF) (2022). Available [here](https://www.england.nhs.uk/publication/decision-support-tool-making-a-decision-about-further-treatment-for-atrial-fibrillation/). | Do nothing vs Medication (rhythm control) vs Left Atrial ablation vs Atrioventricular node ablation & pacemaker | NHS England, UK | English | Print (8 pages) | Before consult |
| 22. Atrial Fibrillation: Should I Have Catheter Ablation? (2024). Available [here](https://decisionaid.ohri.ca/AZsumm.php?ID=1333). | Catheter ablation vs no catheter ablation | Healthwise, USA | English | Online, Print (6 pages) | Before consult |
| 23. Boezemfibrilleren (2023). Available [here](https://www.keuzehulp.info/onvz/af/intro/1). | Do not treat vs Medicines that make the heart beat slower vs His bundle ablation vs Medicines that make the heart rhythm regular vs Electrical cardioversion vs Catheter ablation vs Laparoscopic ablation | Patient+, Netherlands | Dutch | Online (20 pages) | Before consult |
| 24. Atrial fibrillation: How can an irregular heart rhythm be treated? (2022). Available [here.](https://www.informedhealth.org/decision-aid-atrial-fibrillation-how-can-an-irregular-heart-rhythm-be-treated.html) | Slow down heart frequency (rate control), Restore sinus rhythm (electro cardioversion, medication), Maintain sinus rhythm (medication, catheter ablation) | Members of the Health Information department; Quality and efficiency in Health care (IQWiG), Germany | English | Print (6 pages) | Before consult |
| **LVAD and Risk for Stroke (n=4 PtDAs)** | | | | | |
| 25. Deciding Together: A Left Ventricular Assist Device (LVAD) Decision Aid (2018). Available [here](https://www.lvaddecisionaid.com/). | LVAD vs No LVAD | Center for Medical Ethics and Healthy Policy, Baylor College of Medicine, USA | English, Spanish | Online (6-10 pages), Print (69 pages) | Before consult |
| 26. A decision aid for Left Ventricular Assist Device (LVAD): A device for patients with advanced heart failure (2020). Available [here](https://www.patientdecisionaid.org/LVAD/). | LVAD vs No LVAD | University of Colorado School of Medicine, USA | English, French, Spanish | Online, Print (8 pages) and Video (26 min) | Before consult |
| 27. For Patients, families and attendants considering DT treatment** (2021). Available [here](http://www.cardiology.med.osaka-u.ac.jp/wp-content/uploads/2021/12/decision_aids_var.1.pdf). | LVAD vs No LVAD | Osaka University Hospital Heart Centre, Japan | Japanese | Print (32 pages) | Before consult |
| 28. Considering a Left Ventricular Assist Device (LVAD): A Guide for Patients and Caregivers (NR). Available [here](https://www.corhealthontario.ca/Considering-a-Left-Ventricular-Assist-Device-Conversation-Guide-for-Patients-and-Caregivers.pdf). | LVAD vs No LVAD | CorHealth Ontario, Canada | English | Print (21 pages) | Before consult |
| **Aortic Valve Replacement and Risk for Stroke (n=6 PtDAs)** | | | | | |
| 29. Treatment Options for Severe Aortic Stenosis for Patients Deciding Between TAVI And Surgery: For Patients With Low or Intermediate Surgical Risk (2024). Available [here](https://www.cardiosmart.org/docs/default-source/assets/decision-aid/aortic-stenosis-tavr-surgery.pdf?sfvrsn=a59c08f5_3). | TAVR vs SAVR | American College of Cardiology, USA | English | Print (8 pages) | Before consult |
| 30. A decision aid for treatment options for severe aortic stenosis for patients deciding between TAVR and Surgery: For Patients with INTERMEDIATE OR HIGH SURGICAL RISK (2017). Available [here](https://www.jscai.org/cms/10.1016/j.jscai.2022.100025/attachment/6d451938-700e-4d39-86d2-a4d6f84bbe2b/mmc2.pdf). | TAVR vs SAVR | American College of Cardiology, USA | English | Print (7 pages) | Before consult |
| 31. A decision aid for treatment options for severe aortic stenosis (TAVR vs Symptom Management): For Patients With Prohibitive Surgical Risk/Inoperable (2025). Available [here](https://www.cardiosmart.org/topics/aortic-stenosis/assets/decision-aid/choosing-between-tavr-and-symptom-management). | TAVR vs Symptom management (medications) | American College of Cardiology, USA | English | Print (8 pages) | Before consult |
| 32. Severe Aortic Stenosis Decision Aid (2020). Available [here](https://pmc.ncbi.nlm.nih.gov/articles/PMC7160688/figure/hoi190102f1/). | TAVR vs Symptom management (palliative care) | American College of Cardiology, USA | English | Print (1 page) | During consult |
| 33. Nieuwe hartklep (2022). Available [here](https://www.keuzehulp.info/onvz/hartklep/intro/1). | Mechanical vs biological heart valve; Open heart surgery vs Minimally invasive treatment vs Transcatheter Aortic Valve Implantation (TAVI) | Patient+, Netherlands | Dutch | Online (28 pages) | Before consult |
| 34. Hartklep keuzehulp (2017). Available [here](https://hartklep.keuzehulp.nl/inloggen). Create login code to access. | Mechanical vs biological heart valve | ZorgKeuzeLab, Dutch Society for Thoracic Surgery, in collaboration with the Dutch Society for Cardiology, the Dutch Heart Foundation and the Heart & Vascular Group, Netherlands | Dutch | Online (17 pages) | Before consult |
| **Procedures for Coronary Artery Disease (revascularization) and Risk of Stroke (n=7 PtDAs)** | | | | | |
| 35. Coronary Artery Disease: Should I Have Bypass Surgery? (2024). Available [here](https://decisionaid.ohri.ca/AZsumm.php?ID=1318). | Have coronary artery bypass surgery vs. Don't have bypass surgery | Healthwise, USA | English | Online, Print (6 pages) | Before consult |
| 36. Coronary Artery Disease: Should I Have an Angiogram? (2024). Available [here](https://decisionaid.ohri.ca/AZsumm.php?ID=1105). | Angiogram vs No angiogram | Healthwise, USA | English | Online, Print (6 pages) | Before consult |
| 37. Coronary Artery Disease: Should I Have Angioplasty for Stable Angina? (2024). Available [here](https://decisionaid.ohri.ca/AZsumm.php?ID=1202). | Angioplasty vs No angioplasty | Healthwise, USA | English | Online, Print (6 pages) | Before consult |
| 38. Coronary Artery Disease. What treatment would you prefer? (2015). Available [here](https://www.ahajournals.org/action/downloadSupplement?doi=10.1161%2FCIRCOUTCOMES.118.005244&file=circcvqo_circcqo-2018-005244_supp1.pdf). | Medicine alone vs Angioplasty vs Bypass surgery | Healthwise, USA | English | Print (8 pages) | Before consult |
| 39. NSTEMI Decide (2020). Available [here](https://apps.apple.com/us/app/nstemi-decide/id1512358323). | Cardiac catheterization or No cardiac Catheterization | New York University, USA | English, Spanish | App based (12 pages) | Before consult |
| 40. Decision Aid for Stable Ischemic Heart Disease (2025). Available [here](https://www.drugs.com/cg/decision-aid-for-stable-ischemic-heart-disease.html). | Screening for SIHD (EKG, blood test, CT scan, stress test, heart catheterization); Treatment for SIHD (Lifestyle changes, Medicines, PCI, CABG) | Drugs.com, USA | English, Spanish | Print (1 page) | Before consult |
| 41. Decision support tool: making a decision about stable angina (2024). Available [here](https://www.england.nhs.uk/publication/decision-support-tool-making-a-decision-about-stable-angina/). | Medicines only vs Medicines plus  Coronary angioplasty vs Medicines plus CABG | NICE, UK | English | Print (16 pages) | Before consult |
| **Peripheral Arterial Disease and Risk of Stroke (n=2 PtDAs)** | | | | | |
| 42. Show me PAD (2019). Available [here](https://showme-pad.org/). | Exercise therapy vs Claudication medicines vs Angioplasty/Stents/Bypass surgery | Yale University, USA | English | Online (5 pages), Print (6 pages), Video (1:43 min) | Before consult |
| 43. Peripheral Arterial Disease: Should I Have Surgery? (2024). Available [here](https://decisionaid.ohri.ca/AZsumm.php?ID=1108). | Angioplasty or surgery vs Exercise or medicine | Healthwise, USA | English | Online, Print (6 pages) | Before consult |
| **Heart failure and Non-invasive vs Invasive treatments and Risk of Stroke (n=1 PtDA)** | | | | | |
| 44. Heart Failure Decision Aid (2024). Available [here](https://intermountainhealthcare.org/ckr-ext/Dcmnt?ncid=529809926). | Non-Invasive Treatment (Lifestyle changes, medication) vs Invasive Treatment (TAVR, Aortic Valve Replacement Open-Heart Surgery, Pacemaker, Implantable Cardioverter-Defibrillator) vs End-of-Life Care | Intermountain Health, USA | English, Spanish | Print (3 pages) | Unclear |
| **Carotid Artery Stenosis and Risk of Stroke (n= 1 PtDA)** | | | | | |
| 45. Stroke Prevention: Should I Have a Carotid Artery Procedure? (2024). Available [here](https://decisionaid.ohri.ca/AZsumm.php?ID=1157). | Carotid artery procedure vs No carotid artery procedure | Healthwise, USA | English | Online, Print (6 pages) | Before consult |
| **Brain/Mental health as primary condition (n=6)** | | | | | |
| **Mental Health Treatment and Risk of Heart Complications (e.g., arrhythmias, high blood pressure) (n=5 PtDAs)** | | | | | |
| 46. A decision aid for depression: A guide to choosing a treatment for depression: mild cases (2018). Available [here](https://www.frontiersin.org/api/v4/articles/967750/file/Data_Sheet_2.pdf/967750_supplementary-materials_datasheets_2_pdf/1). | Antidepressants, Electroconvulsive therapy, Psychotherapy. | Japanese Agency for Medical Research and Development, Japan | Japanese | Print (32 pages) | Before consult |
| 47. Depression in Older Adults: Choosing an option to improve mood (2018). Available [here](https://www.boitedecision.ulaval.ca/en/box-details/?tx_tmboites_tmboitesmain%5Bclear%5D=1&tx_tmboites_tmboitesmain%5Bboite%5D=58). | Psychotherapy, Physical Activity, Antidepressants, Massage therapy, Light therapy, Watchful waiting | Laval University, Canada | English, French | Online (5 pages), Print (15 pages) | Before consult |
| 48. Zur Entscheidungshilfe: Psychosen (2025). Available [here](https://www.psychenet.de/de/entscheidungshilfen/entscheidungshilfe-psychose/was-ist-eine-psychose/was-ist-eine-psychose-2.html?view=page&layout=index&id=175). | Self-help measures vs Medication vs Psychological Therapies | Psychenet, Germany | German | Online (18 pages) | Before consult |
| 49. A decision aid for ADHD Regarding discontinuation of medications (2021). Available [here](https://pmc.ncbi.nlm.nih.gov/articles/instance/11114424/bin/PCN5-1-e57-s001.pdf). | Continue ADHD medications vs Discontinue ADHD medications | Sleep Drug Group in Psychotropic Drugs Exit Strategy Manual Research Group, Japan | English, Japanese | Print (28 pages) | Before consult |
| 50. Decisions in Recovery: Treatment for Opioid Use Disorder (2016). Available [here](https://library.samhsa.gov/sites/default/files/sma16-4993.pdf). | Methadone, Buprenorphine, Naltrexone | Substance abuse and mental health services administration, USA | English | Print (68 pages) | Before consult |
| **Depression and Cardiovascular Disease (n=1 PtDA)** | | | | | |
| 51. Informed Choices About Depression (2019) – Includes section on Heart Problems and Cardiovascular Disease. Available [here](https://depression.informedchoices.ca/). | Self-Help Treatments, Counseling or Therapy, Medication Treatment, Alternative Treatments | Mobilizing Minds Research Group, Canada | English | Online (5 pages) | Before consult |

Abbreviations: ADHD = Attention-deficit/hyperactivity disorder; CABG = Coronary artery bypass grafting; DOAC = Direct oral anticoagulant; LVAD = Left ventricular assist device; PCI = Percutaneous coronary intervention; PtDA = Patient decision aid; SAVR = Surgical aortic valve replacement; SIHD = Stable ischemic heart disease; TAVI = Transcatheter aortic valve implantation; TAVR = Transcatheter aortic valve replacement

**Translated using Google translate.

Supplemental Table S2. Quality of individual PtDAs: IPDAS checklist for Essential Criteria (n=51 PtDAs) and PEMAT (n=45)

| **ID** | **1. Best available evidence** | **2. How users were involved** | **3. Features of options balanced** | **4. Funding source** | **5. Complete citations** | **6. Publication date** | **7. Update policy** | **Total out of 7** | **PEMAT-P** | | **PEMAT-AV** | |
| --- | --- | --- | --- | --- | --- | --- | --- | --- | --- | --- | --- | --- |
|  |  |  |  |  |  |  |  |  | **Understandability (0-100)** | **Actionability (0-100)** | **Understandability (0-100)** | **Actionability (0-100)** |
| 1 | ✓ | ✓ (HCPs only) | ✓ | ✓ | ✓ | ✓ | ✓ | 7 | 93.8 | 83.3 | N/A | N/A |
| 2 | ✓ |  |  |  | ✓ | ✓ |  | 3 | 84.6 | 40.0 | N/A | N/A |
| 3 | ✓ |  |  |  | ✓ | ✓ |  | 3 | 84.6 | 60.0 | N/A | N/A |
| 4 | ✓ | ✓ (Patients + HCPs) | ✓ |  | ✓ | ✓ | ✓ | 6 | 81.2 | 66.7 | N/A | N/A |
| 5 | ✓ | ✓ (HCPs only) | ✓ | ✓ | ✓ | ✓ | ✓ | 7 | 92.9 | 66.7 | N/A | N/A |
| 6 | ✓ | ✓ (HCPs only) | ✓ | ✓ | ✓ | ✓ | ✓ | 7 | 92.9 | 83.3 | N/A | N/A |
| 7 | ✓ | ✓ (Patients + HCPs) | ✓ |  | ✓ | ✓ | ✓ | 6 | 81.2 | 50.0 | N/A | N/A |
| 8 |  |  | ✓ |  |  |  |  | 1 | 82.4 | 83.3 | N/A | N/A |
| 9 | ✓ | ✓ (Patients + HCPs) | ✓ | ✓ | ✓ | ✓ | ✓ | 7 | 52.9 | 0.0 | N/A | N/A |
| 10 | ✓ | ✓ (HCPs only) | ✓ | ✓ | ✓ | ✓ | ✓ | 7 | 92.9 | 83.3 | N/A | N/A |
| 11 | ✓ | ✓ (Patients + HCPs) | ✓ | ✓ | ✓ |  |  | 5 | 81.2 | 66.7 | N/A | N/A |
| 12 | ✓ | ✓ (HCPs only) | ✓ | ✓ |  |  |  | 4 | 93.8 | 100.0 | 100.0 | 100.0 |
| 13 | ✓ |  | ✓ |  | ✓ |  |  | 3 | 57.1 | 57.1 | N/A | N/A |
| 14 | ✓ | ✓ (Patients + HCPs) | ✓ | ✓ | ✓ |  |  | 5 | 76.5 | 57.1 | N/A | N/A |
| 15 | ✓ | ✓ (HCPs only) | ✓ | ✓ | ✓ | ✓ |  | 6 | 87.5 | 100.0 | N/A | N/A |
| 16 |  | ✓ (Patients + HCPs) | ✓ | ✓ |  | ✓ | ✓ | 5 | 82.3 | 83.3 | 92.3 | 100.0 |
| 17 |  | ✓ (Patients + HCPs) | ✓ | ✓ |  | ✓ | ✓ | 5 | 82.3 | 83.3 | 92.3 | 100.0 |
| 18 | ✓ | ✓ (HCPs only) | ✓ | ✓ | ✓ | ✓ | ✓ | 7 | 78.6 | 66.7 | N/A | N/A |
| 19 | ✓ |  | ✓ |  | ✓ | ✓ | ✓ | 5 | 85.7 | 33.3 | N/A | N/A |
| 20 |  |  | ✓ | ✓ |  | ✓ | ✓ | 4 | 88.2 | 100.0 | 100.0 | 75.0 |
| 21 | ✓ | ✓ (Patients + HCPs) | ✓ | ✓ | ✓ | ✓ |  | 6 | 94.1 | 83.3 | N/A | N/A |
| 22 | ✓ | ✓ (HCPs only) | ✓ | ✓ | ✓ | ✓ | ✓ | 7 | 100.0 | 83.3 | N/A | N/A |
| 23 | ✓ | ✓ (Patients + HCPs) | ✓ | ✓ | ✓ | ✓ | ✓ | 7 | Not English | |  |  |
| 24 | ✓ | ✓ (Patients + HCPs) | ✓ | ✓ |  | ✓ | ✓ | 6 | 85.7 | 66.7 | N/A | N/A |
| 25 | ✓ | ✓ (HCPs only) | ✓ | ✓ | ✓ | ✓ | ✓ | 7 | 82.4 | 83.3 | N/A | N/A |
| 26 | ✓ | ✓ (Patients + HCPs) | ✓ | ✓ | ✓ | ✓ | ✓ | 7 | 88.2 | 83.3 | 100.0 | 100.0 |
| 27 | ✓ | ✓ (Patients + HCPs) | ✓ | ✓ | ✓ | ✓ | ✓ | 7 | Not English | |  |  |
| 28 | ✓ | ✓ (Patients + HCPs) | ✓ |  | ✓ |  |  | 4 | 88.2 | 66.7 | N/A | N/A |
| 29 |  |  | ✓ | ✓ |  | ✓ | ✓ | 4 | 88.2 | 83.3 | N/A | N/A |
| 30 | ✓ | ✓ (HCPs only) | ✓ | ✓ | ✓ | ✓ | ✓ | 7 | 100.0 | 66.7 | N/A | N/A |
| 31 | ✓ | ✓ (HCPs only) | ✓ | ✓ | ✓ | ✓ | ✓ | 7 | 94.1 | 83.3 | N/A | N/A |
| 32 | ✓ | ✓ (Patients + HCPs) | ✓ | ✓ |  |  |  | 4 | 80.0 | 50.0 | N/A | N/A |
| 33 | ✓ | ✓ (Patients + HCPs) | ✓ | ✓ | ✓ | ✓ | ✓ | 7 | Not English | |  |  |
| 34 | ✓ | ✓ (Patients + HCPs) | ✓ | ✓ | ✓ | ✓ | ✓ | 7 | Not English | |  |  |
| 35 | ✓ | ✓ (HCPs only) | ✓ | ✓ | ✓ | ✓ | ✓ | 7 | 100.0 | 83.3 | N/A | N/A |
| 36 | ✓ | ✓ (HCPs only) | ✓ | ✓ | ✓ | ✓ | ✓ | 7 | 92.9 | 66.7 | N/A | N/A |
| 37 | ✓ | ✓ (HCPs only) | ✓ | ✓ | ✓ | ✓ | ✓ | 7 | 100.0 | 83.3 | N/A | N/A |
| 38 | ✓ | ✓ (HCPs only) | ✓ | ✓ | ✓ | ✓ | ✓ | 7 | 94.1 | 83.3 | N/A | N/A |
| 39 | ✓ | ✓ (HCPs only) | ✓ | ✓ | ✓ | ✓ |  | 6 | 94.1 | 66.7 | N/A | N/A |
| 40 | ✓ | ✓ (HCPs only) | ✓ |  |  | ✓ |  | 4 | 83.3 | 40.0 | N/A | N/A |
| 41 | ✓ | ✓ (Patients + HCPs) | ✓ |  | ✓ | ✓ | ✓ | 6 | 88.2 | 83.3 | N/A | N/A |
| 42 | ✓ | ✓ (Patients + HCPs) | ✓ | ✓ | ✓ | ✓ |  | 6 | 58.8 | 83.3 | 66.7 | 100.0 |
| 43 | ✓ | ✓ (HCPs only) | ✓ | ✓ | ✓ | ✓ | ✓ | 7 | 100.0 | 100.0 | N/A | N/A |
| 44 |  |  | ✓ |  |  | ✓ |  | 2 | 62.5 | 33.3 | N/A | N/A |
| 45 | ✓ | ✓ (HCPs only) | ✓ | ✓ | ✓ | ✓ | ✓ | 7 | 100.0 | 100.0 | N/A | N/A |
| 46 | ✓ | ✓ (Patients + HCPs) | ✓ | ✓ | ✓ | ✓ | ✓ | 7 | Not English | |  |  |
| 47 | ✓ | ✓ (Patients + HCPs) | ✓ | ✓ | ✓ | ✓ | ✓ | 7 | 100.0 | 93.8 | N/A | N/A |
| 48 | ✓ | ✓ (Patients + HCPs) | ✓ |  | ✓ | ✓ | ✓ | 6 | Not English | |  |  |
| 49 | ✓ | ✓ (Patients + HCPs) | ✓ | ✓ | ✓ | ✓ | ✓ | 7 | 82.4 | 100.0 | N/A | N/A |
| 50 | ✓ | ✓ (Patients + HCPs) | ✓ | ✓ | ✓ | ✓ |  | 6 | 85.7 | 100.0 | N/A | N/A |
| 51 | ✓ | ✓ (Patients + HCPs) | ✓ | ✓ | ✓ | ✓ |  | 6 | 84.6 | 66.7 | N/A | N/A |
| Total | 45 | 43 | 49 | 39 | 41 | 44 | 34 |  |  |  |  |  |

Abbreviations: HCPs = Healthcare Providers; N/A = Not applicable

Table Legend:

1. Based on best available evidence that is, where possible, directly applicable to the patients and clinicians using it

2. Describes how potential users were involved in steps of designing, developing and/or refining a prototype

3. Shows negative/positive features of options in a balanced manner (e.g., neutral, unbiased, non-directive, complete)

4. Reports where the money came from to develop the PDA and it is clearly stated (e.g., plain language, prominent)

5. Provides complete citations to evidence selected

6. Provides a production or publication date

7. Provides information about the proposed update policy (or available supporting document)

Supplemental Table S3. PROGRESS-Plus full details (n=51)

| **ID** | **P** | **Ra** | **O** | **G** | **Re** | **E** | **SES** | **SC** | **Plus (1)** | **Plus (2)** | **Plus (3)** | **Total** |
| --- | --- | --- | --- | --- | --- | --- | --- | --- | --- | --- | --- | --- |
| 1 |  | ✓  race |  | ✓  gender |  |  |  |  | ✓  age |  |  | 3 |
| 2 |  | ✓  race |  | ✓  sex |  |  |  |  | ✓  age - family history (<55y M; <65y F) |  |  | 3 |
| 3 |  |  |  | ✓  sex |  |  |  |  | ✓  age (≥80y) |  |  | 2 |
| 4 |  | ✓  ethnicity |  | ✓  sex |  |  |  |  | ✓  age |  |  | 3 |
| 5 |  | ✓  race or ethnic group |  | ✓  gender |  |  |  |  | ✓  age (<40y; >60-70y) |  |  | 3 |
| 6 |  |  |  | ✓  gender |  |  | ✓  costs |  | ✓  age (>65y); family history (<55y M; <65y F) |  |  | 3 |
| 7 |  | ✓  Black  African or African–  Caribbean family origin |  | ✓  sex |  |  |  |  | ✓  age |  |  | 3 |
| 8 |  | ✓ Indigenous |  | ✓  sex |  |  |  |  | ✓  age |  |  | 3 |
| 9 |  |  |  |  |  |  |  |  | ✓  age |  |  | 1 |
| 10 |  |  |  | ✓  sex |  |  | ✓  costs |  | ✓  age (>65y) |  |  | 3 |
| 11 |  |  |  | ✓  sex |  |  | ✓  costs |  | ✓  age |  |  | 3 |
| 12 |  |  |  | ✓  sex |  |  | ✓  costs |  | ✓  age (65-74y, ≥75y) |  |  | 3 |
| 13 |  |  |  | ✓  sex |  |  |  |  | ✓  age (65-74y, ≥75y) |  |  | 2 |
| 14 |  |  |  | ✓  sex |  |  |  | ✓  family support | ✓  age (65-74y, ≥75y) |  |  | 3 |
| 15 |  |  |  |  |  |  | ✓  costs |  | ✓  age (≤64; 65-74y; ≥75y |  |  | 2 |
| 16 |  |  |  |  |  |  | ✓  costs |  | ✓  age |  |  | 2 |
| 17 |  |  |  |  |  |  | ✓  costs |  | ✓  age |  |  | 2 |
| 18 |  |  |  |  |  |  | ✓  costs, health insurance |  | ✓  age |  |  | 2 |
| 19 |  |  |  | ✓  sex |  |  |  |  | ✓  age (65-74y; ≥75y; >65) |  |  | 2 |
| 20 |  |  |  |  |  |  | ✓  costs |  | ✓  age |  |  | 2 |
| 21 |  |  | ✓  ability to work |  |  |  |  |  |  |  |  | 1 |
| 22 |  |  |  |  |  |  |  |  | ✓  age |  |  | 1 |
| 23 |  |  |  |  |  |  | ✓  costs | ✓  family support | ✓  age (<75y) |  | ✓  pregnancy | 4 |
| 24 |  |  |  |  |  |  |  |  | ✓  age |  |  | 1 |
| 25 |  |  | ✓  time off work |  | ✓ |  |  | ✓  family support | ✓  age |  |  | 4 |
| 26 |  |  |  |  |  |  | ✓  costs | ✓  family support |  |  |  | 2 |
| 27 |  |  | ✓  return to work | ✓  sex |  | ✓  return to school | ✓  costs, income, insurance benefits, disability pension | ✓  family support | ✓  age |  |  | 6 |
| 28 | ✓  accommodation near hospital |  | ✓  time off work |  |  |  | ✓  costs | ✓  family support |  |  |  | 4 |
| 29 |  |  |  |  |  |  |  |  |  |  |  | 0 |
| 30 |  |  |  |  |  |  |  |  |  |  |  | 0 |
| 31 |  |  |  |  |  |  |  |  |  |  |  | 0 |
| 32 |  |  |  |  |  |  |  |  |  |  |  | 0 |
| 33 |  |  | ✓  time off work | ✓  sex |  |  |  | ✓  family support | ✓  age, poor mental health |  | ✓  pregnancy | 5 |
| 34 | ✓ |  |  | ✓  gender |  |  |  |  | ✓  age |  | ✓  pregnancy | 4 |
| 35 |  |  |  |  |  |  |  |  | ✓  age |  |  | 1 |
| 36 |  |  |  |  |  |  | ✓  costs |  | ✓  age |  |  | 2 |
| 37 |  |  |  |  |  |  |  |  | ✓  age |  |  | 1 |
| 38 |  |  | ✓  return to work |  |  |  |  |  |  |  |  | 1 |
| 39 |  |  |  |  |  |  |  |  | ✓  age (≥75y) |  |  | 1 |
| 40 |  |  |  |  |  |  | ✓  insurance coverage |  | ✓  age |  |  | 2 |
| 41 |  |  |  |  |  |  |  |  | ✓  age |  |  | 1 |
| 42 |  |  | ✓  time off work |  |  |  | ✓  costs |  | ✓  age |  |  | 3 |
| 43 |  |  | ✓  limited ability to work |  |  |  |  |  |  |  |  | 1 |
| 44 |  |  |  | ✓  sex | ✓  Emotional support (e.g. chaplain) |  |  | ✓  social support |  |  |  | 3 |
| 45 |  |  |  |  |  |  |  |  | ✓  age (>70y) |  |  | 1 |
| 46 |  |  | ✓  interference with work | ✓  sex |  | ✓  interference with school |  | ✓  family support; relationship dynamics | ✓  age (30-50y; <24y) |  | ✓  stressful life events; pregnancy | 6 |
| 47 |  |  |  |  |  |  | ✓  costs | ✓  relationship dynamics | ✓  age, stigma |  | ✓  stressful life events | 4 |
| 48 |  |  |  | ✓  gender |  |  |  | ✓  relationship dynamics | ✓  age (12-29y) |  | ✓  unstable life periods | 4 |
| 49 |  |  | ✓  managing tasks at work | ✓  sex |  | ✓  managing tasks at school | ✓  costs | ✓  family support; relationship dynamics | ✓  age (<12y; <18y) |  | ✓  pregnancy | 7 |
| 50 | ✓  live close to treatment centre; stable housing |  | ✓  keep or get job; time off work; disclosure to employer | ✓  sex; gender | ✓  faith-based social support | ✓  go or stay in school | ✓  costs | ✓  family support; relationship dynamics; social support | ✓  (<18y); stigma/discrimination | ✓  physical or sexual abuse | ✓  pregnancy; post-release from prison | 10 |
| 51 |  |  | ✓  interference with work | ✓  gender |  | ✓  interference with school | ✓  costs, insurance coverage | ✓  family support; relationship dynamics | ✓  age |  | ✓  stressful life events | 7 |
| Total | 3 | 6 | 12 | 23 | 3 | 5 | 20 | 14 | 41 | 1 | 9 | 137 |

Table Legend:

**P** = Place of residence - where someone lives (e.g. the country, region, city, community and their characteristics, or urban vs. rural settings)

**R**a = Race/ethnicity/culture/language

**O** = Occupation - e.g. unemployment, underemployment, informal employment, and unsafe working condition, as well as type of occupation

**G** = Gender/sex

**R**e = Religion

**E** = Education

**S**ES = Socioeconomic status

**S**C = Social capital - e.g. social relationships and networks

Plus (1) = personal characteristics associated with discrimination (e.g. age, disability)

Plus (2) = features of relationships (e.g. smoking parents, excluded from school)

Plus (3) = time-dependent relationships (e.g. leaving the hospital, respite care, other instances where a person may be temporarily at a disadvantage)
